# Cyanovirin-N Binding to *N*-Acetyl-d-glucosamine Requires Carbohydrate-Binding Sites on Two Different Protomers

**DOI:** 10.1021/acs.biochem.4c00113

**Published:** 2024-04-09

**Authors:** Irene Maier, Georg Kontaxis, Christian Zimmermann, Christoph Steininger

**Affiliations:** † Department of Environmental Health Sciences, Fielding School of Public Health, University of California, Los Angeles, 650 Charles E. Young Dr. South, Los Angeles, California 90095, United States; ‡ Department of Internal Medicine I, Medical University of Vienna, Waehringer Guertel 18-20, Vienna A-1090, Austria; § Department of Computational and Structural Biology, Max Perutz Laboratories, University of Vienna, Campus Vienna Biocenter 5, Vienna 1030, Austria; ∥ Institute of Chemical, Environmental and Bioscience Engineering, TU Wien, Gumpendorfer Strasse 1a, Wien 1060, Austria

## Abstract

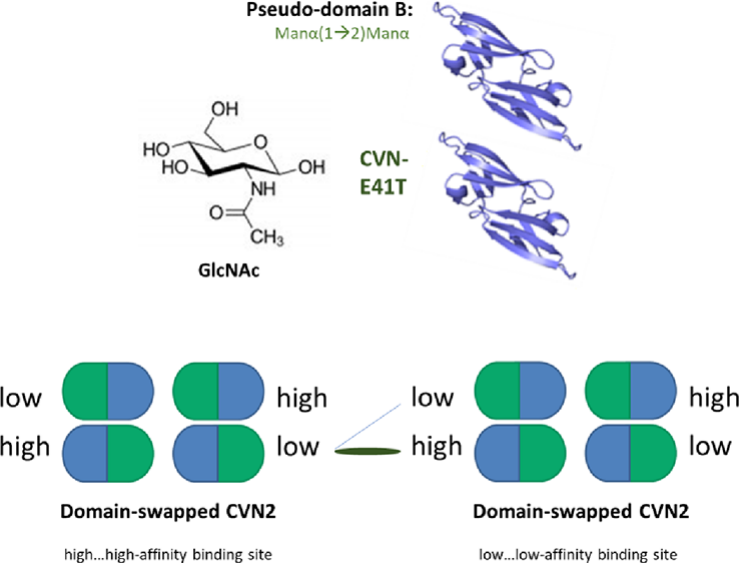

Cyanovirin-N (CV-N) binds high-mannose oligosaccharides on enveloped viruses with two carbohydrate-binding sites, one bearing high affinity and one low affinity to Manα(1–2)Man moieties. A tandem repeat of two CV-N molecules (CVN2) was tested for antiviral activity against human immunodeficiency virus type I (HIV-1) by using a domain-swapped dimer. CV-N was shown to bind *N*-acetylmannosamine (ManNAc) and *N*-acetyl-d-glucosamine (GlcNAc) when the carbohydrate-binding sites in CV-N were free to interact with these monosaccharides independently. CVN2 recognized ManNAc at a *K*_d_ of 1.4 μM and bound this sugar in solution, regardless of the lectin making amino acid side chain contacts on the targeted viral glycoproteins. An interdomain cross-contacting residue Glu41, which has been shown to be hydrogen bonding with dimannose, was substituted in the monomeric CV-N. The amide derivative of glucose, GlcNAc, achieved similar high affinity to the new variant CVN-E41T as high-mannose *N*-glycans, but binding to CVN2 in the nanomolar range with four binding sites involved or binding to the monomeric CVN-E41A. A stable dimer was engineered and expressed from the alanine-to-threonine-substituted monomer to confirm binding to GlcNAc. In summary, low-affinity binding was achieved by CVN2 to dimannosylated peptide or GlcNAc with two carbohydrate-binding sites of differing affinities, mimicking biological interactions with the respective *N*-linked glycans of interest and cross-linking of carbohydrates on human T cells for lymphocyte activation.

## Introduction

CV-N is a small cyanobacterial lectin that binds specifically to high-mannose oligosaccharide modifications on viral envelope spike proteins.^1,2^ The lectin is binding viruses near the receptor-binding site or blocking the interaction with CD4, thereby preventing virus internalization and dissemination.^2−4^ Binding of high-mannose glycan moieties was resolved in CV-N with two carbohydrate-binding sites of differing affinities located on opposite protein protomers.^5−8^ The total number of carbohydrate-binding sites, increased by oligomerization, was found to enhance CV-N’s broad neutralization capacity on HIV-1 subtypes,^9^ whereas CV-N was also found active against other retroviruses^3^ and beta-coronaviruses.^10^ Multiple studies investigated the trapped fold of domain-swapped dimer in the CVN2 crystal structures, linking domain A and B′ and domain B with A′ to form new monomeric units.^5,9^ The type of *N*-glycans and the way they are positioned on viral spikes, rather than the actual number of *N*-linked glycosylation sites,^10,11^ or the molecular imprint of chemically synthesized and mannosylated peptides,^12^ were relevant for binding these ligands to CV-N and CVN2.^11^

Selective binding of α(1–2)-linked di- or trimannose units on the terminal arms of the branched Man-8 and Man-9 structures^7^ on HIV gp120 comprised two CVN2 binding sites^1,12^ on two domains B,^13^ or one on domain B and one on domain A, thereby reaching nanomolar binding affinities.^7−9,14^ The thermodynamics in sugar binding to CV-N was measured to linear trimannoside and nonamannoside with a two-site model and hexamannoside with a one-site model to determine increasing affinity from tri- to nonamannoside to CV-N.^7^ Manα1 → 2Man moieties were bound at the CV-N deeper high-affinity binding pocket^13,15^ with micromolar affinity for trimannoside and hexamannoside, but a nanomolar dissociation constant (*K*_d_) for nonamannoside.^7^ A model for the binding of dimannose with avidity and high-affinity sites of stabilized monomeric P51G-CV-N,^5^ in which carbohydrate-binding was examined on domains B and B′, was studied by Fromme.^16^ Structural analyses demonstrated that Glu41 and Arg76 were involved in binding carbohydrates,^5,15^ where Glu41 was an intramolecular domain linker and Arg76 a flexible polar residue allowing for allosteric interactions^15^ and hydrogen bonding with dimannose linked to triazole near the second disulfide bonds in the CVN2 molecule.^6,12^ The mechanism by which mannosylated residues and *N*-acetyl-d-glucosamine (GlcNAc) inhibited transcytosis of HIV-1 viruses by interaction with CV-N is hardly understood.^17^ The monosaccharide GlcNAc, as recognized by various antiviral mannose-specific lectins,^18−20^ was specifically bound by chitinase-like lectin against alphavirus^21^ and carbohydrate-binding proteins to *N*-glycans on the surface of enveloped viruses.^22^ Moreover, a significant increase in interferon-γ was observed solely in natural killer (NK)1.1 expressing cell populations upon interaction with GlcNAc-coated particles along with high levels of immunoglobulin (Ig)G2a, resulting in antibody-dependent cellular cytotoxicity (ADCC) enhancement and antibody response to melanoma.^23^ While the role of GlcNAc-specific binding of carbohydrate-binding proteins is mostly antiviral in its character, GlcNAc- (or NAG) receptors other than lectins were associated with bacterial host–pathogen interactions.^24^

Binding of plant lectins to hemagglutinin (HA) or spike protein^20^ led the search for new targets to screenings among small sugars, which were part of the bacterial cell wall, or terminal units of the complex-type oligosaccharides exposed at the surface of human cells.^25^ The assays explored the selectivity and binding specificity of the agents to the oligomannosides on envelope glycoproteins^20^ and used CV-N to target carbohydrates on certain entities outside the receptor-binding domain (RBD).^10^ Neutralizing epitopes on human coronavirus OC43, a coronavirus which emerged long before severe acute respiratory syndrome coronavirus 2 (SARS-CoV-2), were detected to block the sialoglycan binding domain.^25^ Concomitant binding of free monosaccharides was examined,^18^ which did not trigger the fusogenic conformational changes like binding to antibody-targeted epitopes.^10,26^ Previously, we showed binding of CVN2 to viral spikes and addressed the formation of clusters of pseudoantibodies and stoichiometry upon binding with CVN2.^11,27^ In this study, binding between GlcNAc or *N*-acetylmannosamine (ManNAc) by *de novo* hydrogen bonding through binding-site variant CVN-E41T^28^ and four-site domain-swapped dimer CVN2L0 is evaluated using isothermal titration calorimetry (ITC) and saturation transfer difference (STD)-NMR spectroscopy.

## Materials and Methods

### Protein Expression and Purification

The CV-N gene construct contained an *N*-terminal 6-histidine purification tag followed by a Factor Xa protease cleavage site and was subcloned into the NdeI and *Bam*HI sites of pET11a, as described previously.^9^ Mutant CVN-E41A was expressed and tested for dimannose binding,^29^ while CVN-E41T was generated by alanine to threonine substitution. The mutant was further expressed with a tandem repeat of two sequences. The expression of WT CV-N, dimeric CVN2L0, and binding-site mutant CVN-E41T was induced with 1 mM isopropyl β-d-1-thiogalactopyranoside (IPTG) in BL21 (DE3) *Escherichia coli* cells in LB including ampicillin and additives, such as 10 mM MgCl_2_, 10 mM MgSO_4_, and 20 mM glucose, at 20 °C. The cells were grown overnight and harvested by centrifugation at 4000*g* and 4 °C for 15 min, and the supernatant was discarded. The cell pellet was suspended in phosphate-buffered saline (PBS) buffer, recentrifuged, and resuspended in 10 mL of lysis buffer [50 mM NaH_2_PO_4_, 300 mM NaCl, 2% Triton X-100, 500 ng/mL lysozyme, 1 mM phenylmethylsulfonylfluoride (PMSF), 1 mM dithiothreitol, 1 mM MgCl_2_, pH 8] for an incubation of 1 h at 37 °C. The mixture was subjected to two freeze–thaw cycles (−80 °C) before soluble and insoluble fractions were separated by centrifugation at 4000*g* and 4 °C for 15 min. The solubilized CV-N, CVN2, CVN-E41T, and mutant CVN-E41T dimer were then purified under native conditions on a His-Select Ni^2+^ affinity gel (Sigma-Aldrich, St. Louis, Missouri, USA) in 14 mL Falcon tubes. The Ni-NTA gel bound and resuspended recombinant CV-N in buffer solutions with 20 mM imidazole and 250 mM imidazole, respectively. The eluate was transferred onto PD MidiTrap G-10 (Sigma-Aldrich) single-use columns for buffer exchange and verified as monomer or dimer via sodium dodecyl sulfate polyacrylamide gel electrophoresis (SDS-PAGE) as described in ref (27). Protein solutions were put to the final working concentration using Amicon tubes (Merck) with a 10 kDa cutoff filter and centrifugation (Beckman Coulter Cooler Allegra X-30R centrifuge at 4500*xg*, 4 °C). The protein concentration was determined at 280 nm by using a NanoDrop UV–vis 2000c spectrophotometer (Thermo Fisher Scientific, Waltham, Massachusetts, USA).

### Isothermal Titration Calorimetry

Calorimetric titrations were carried out using an ITC200 device (Malvern Panalytical, Malvern, UK). The concentration of mutant E41T dimer and monomeric variants CVN-E41T or CVN-E41A was 200 μM titrant of these proteins to the ligands ManNAc and GlcNAc in the cell. For the CV-N monomer experiments, 607 μM CV-N was titrated into a calorimetry cell containing 6 μM ManNAc, or 295 μM CV-N into 6 μM GlcNAc (both sugars were obtained from Sigma-Aldrich). The protein concentrations were 44 μM (304 μM) CVN2L0 and 465 μM CVN-E41T in the ManNAc experiments, respectively, and determined to be between 200 and 300 μM for the GlcNAc experiment. Aliquots (2 μL) of CV-N titrant were injected at 2 min intervals into the sugar solution in the calorimetry cell with a total of 19 injections from a stirring syringe (operated at 750 rpm). Controls were prepared with identical amounts of titrant injected into PBS buffer at pH 7.4 at 298 K, and control values were subtracted from the results of the actual experiments. The values for enthalpy, binding affinity, and stoichiometry from the binding curve and the free energy and entropy of interaction were calculated using Microcal Origin v7.0 (OriginLab, Northampton, Massachusetts, USA) according to the standard expressions: Δ*G* = −RTln *K*a, and Δ*G* = Δ*H*–TΔ*S*. The data was achieved in triplicate measurement for calculating SEM.

### STD-NMR Experiments

NMR experiments were performed using a Bruker 600 MHz spectrometer, and STD spectra were obtained with −0.05 ppm as irradiation frequency and 2.94 s of saturation time. An NMR sample of CV-N, its mutant, and dimer were prepared in 100 mM PBS, pH 7.0, each added to the carbohydrate solutions in phosphate buffer for a final protein concentration of 100 μM and a ratio of carbohydrate/CV-N = 10:1.

## Results and Discussion

### ManNAc Bound CVN2 at Two Low-Affinity Binding Sites

**Figure 1:**
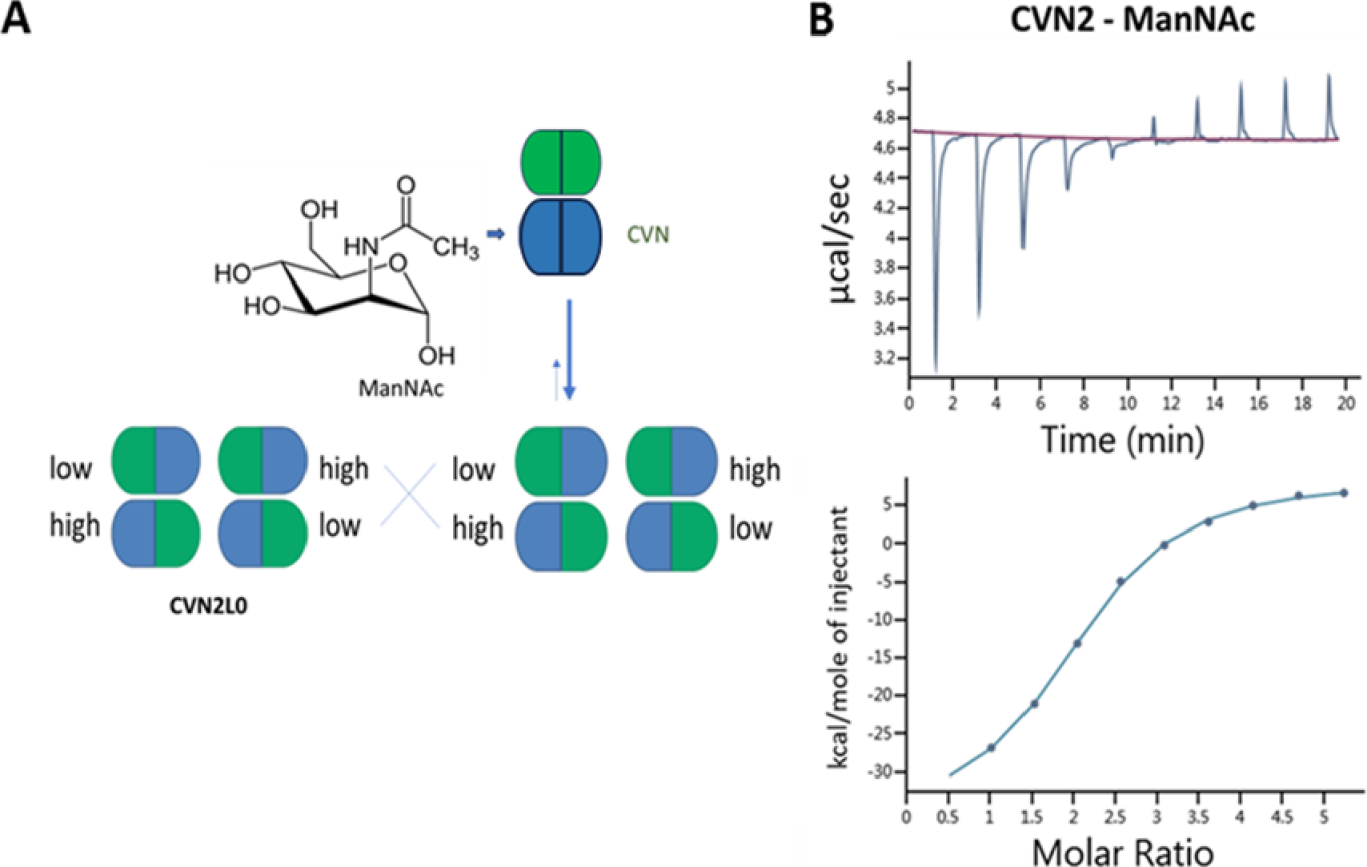
Binding affinity of CVN2 to ManNAc. (A) Schematic representation of monomeric CV-N vs domain-swapped CVN2 binding to carbohydrates, i.e., ManNAc. Green halves represent low-affinity binding domain A, and blue halves represent high-affinity binding domain B. (B) ITC data showing the binding of CVN2 to ManNAc. Thermograms are shown, and binding curves are fitted to the one-set of sites binding model. *T* = 298 K.

To determine CV-N’s binding specificity to high-mannose *N*-glycans, bivalent binding with dimannose linked to propargylglycine in a 12mer peptide from HA was measured by surface plasmon resonance (SPR) spectroscopy^12^ and herein the binding of CVN2 to monosaccharides GlcNAc and ManNAc by ITC. To confirm the stoichiometry of three molecules CV-N that bound Man-9^13,14^ and the amide derivate of mannose (Figure 1), the binding of ManNAc was measured to monomeric CV-N (Table S2) and dimeric CVN2 (Figure 1A,B). Multiple binding sites (four) with higher affinity were detected for two CVN2.^5,8^

**Figure 2:**
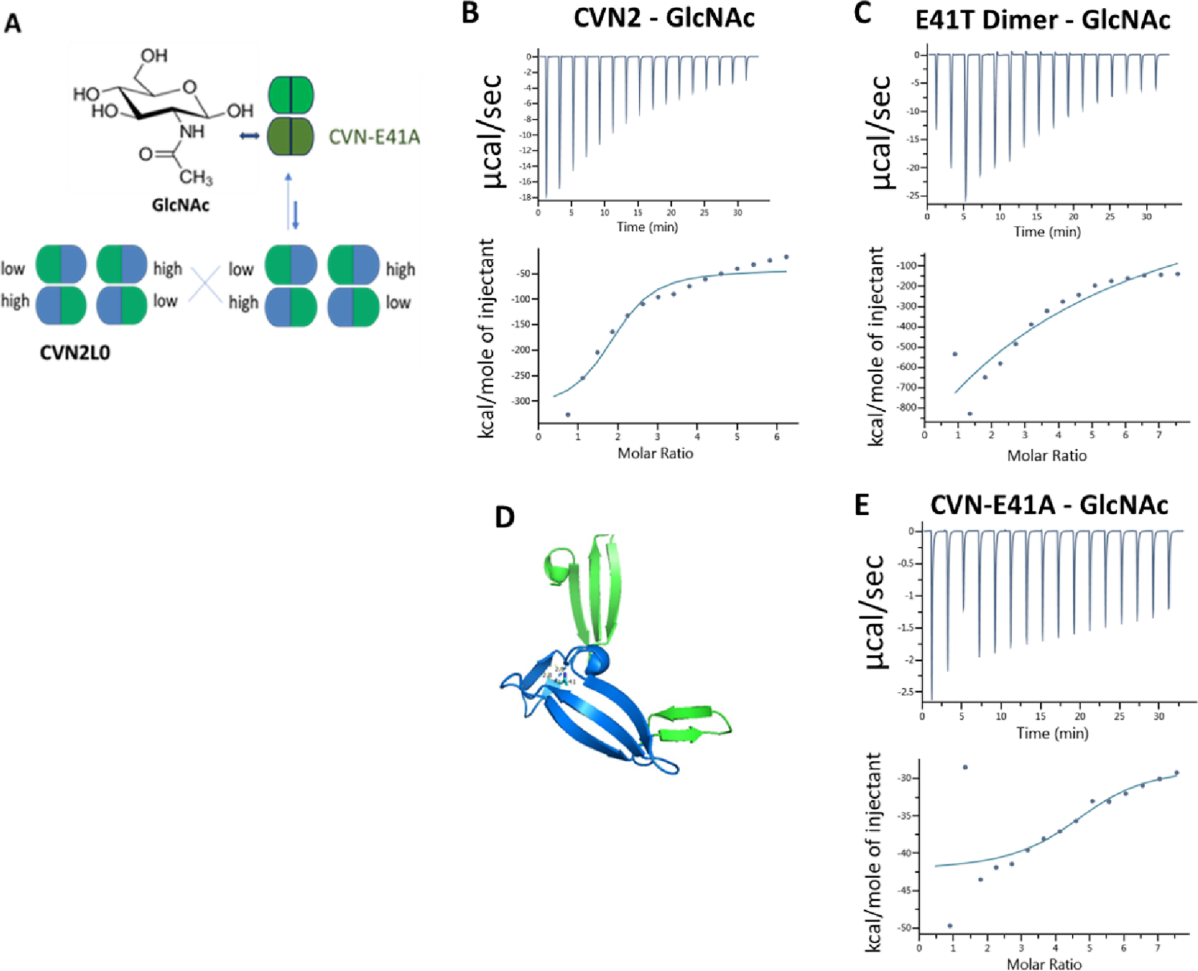
ITC data showing the binding of CVN2, mutant E41T dimer, and CVN-E41A to GlcNAc. (A) Schematic representation of monomeric CVN-E41A and dimeric CVN2 binding to GlcNAc. (B) Binding affinity of CVN2 to GlcNAc. Thermogram is shown, and the binding curve fitted to the one-set of sites model. (C) Mutant E41T dimer binds to GlcNAc. Thermogram is shown, and binding analyzed based on one-set of sites binding model. *T* = 298 K. Ligand in cell. (D) 3D X-ray structure from CV-N (PDB ID:3S3Y) with mutated Ala41 shown as a stick. High-affinity carbohydrate-binding site (AAs 40–89) in blue; low-affinity carbohydrate-binding site (AAs 1–39, 90–101) in green. (E) Binding of CVN-E41A to GlcNAc. Thermogram is shown, and binding analyzed based on one-set of sites binding model. *T* = 298 K. The experiments were performed in triplicate.

Specific carbohydrate–protein interactions with polar and charged residues were delineated from the solution structure with Manα1 → 2Man disaccharide bound to domain B.^5^ Dimerization of CV-N, by increasing the number of accessible binding sites and symmetry in the actual scaffolds, was found essential for enhanced affinities and avidity to surface envelope glycoproteins.^8,9^ A low-affinity binding site was exposed and calculated to bind ManNAc with micromolar concentrations of CV-N and CVN2 (Figure S1A and Figure 1B). Interestingly, GlcNAc was recognized by a single type of carbohydrate-binding sites, which represented a two-site state in the dimer and binding with homologous carbohydrate-binding sites. The binding enthalpies were measured at −34.8 to −80 kcal/mol for both ManNAc and GlcNAc (Table S2), and Gibbs energies for CVN2 binding to ManNAc (−7.0 kcal/mol, Table 1) and GlcNAc (−8.30 kcal/mol, Table 2). Although titration of CVN2 to ManNAc revealed high-affinity binding sites in the low-nanomolar range and a low-affinity carbohydrate-binding site, the binding of ManNAc with two homologous sites at the 10-fold higher receptor concentration was achieved at a *K*_d_ of 1.4 μM (Figure 1B and Table 1).^30^ Our data confirmed binding to domain A, located near the termini, which bound to Manα1 → 2Man disaccharide with about the same *K*_d_ of 1.5 μM.^13^ If comparing equilibrium *K*_d_ of approximately 140 nM for Manα1 → 2Man moieties at the CV-N deeper high-affinity binding pocket^13,15^ with *K*_d_s for ManNAc and GlcNAc (Figure S1A,B), the specific binding of CVN2 was measured for ManNAc (Figure 1B and Table 1). By contrast, neither the interaction between GlcNAc and CV-N (Table S2) nor between GlcNAc and CVN2L0, a tandem-linked dimeric CVN2 with zero-linker length,^9^ showed selective binding via the high-affinity site (Figure 2A,B) but a biphasic interaction with GlcNAc. The binding of ManNAc in comparison with GlcNAc was observed for two sites different in affinities to the monosaccharide ManNAc (Table S2). A previously reported binding model suggested cross-linking of two homologous CVN2 carbohydrate-binding sites by divalent ligands, and concentration-dependent interaction with both sites, thereby forming clusters of virus particle agglutination.^5^

**Table 1:** Thermodynamic Binding Parameters for the Binding of CVN2 to ManNAc

	**enthalpy Δ*H***(kcal/mol)	**entropy *T*Δ*S***(kcal/mol)	**free energy Δ*G***(kcal/mol)	**affinity *K*_d_ [μM]**	**stoichiometry**
**CVN2:ManNAc**	–68.3 ± 20.3	–61.2 ± 21.08	–7.0 ± 1.02	1.4 ± 0.139	1.96 ± 0.024

**Table 2:** Calculated Energies of Interaction, Affinities, and Stoichiometry for the Binding of CVN2, Mutant E41T Dimer, and CVN-E41A to GlcNAc

	**enthalpy Δ*H***(kcal/mol)	**entropy *T*Δ*S***(kcal/mol)	**free energyΔ*G***(kcal/mol)	**affinity *K*_d_ [μM]**	**stoichiometry**
**CVN2:GlcNAc**	–69.9 ± 17.2	–61.7 ± 17	–8.30 ± 0.18	0.852 ± 0.235	4.33 ± 0.335
**mutant E41T- Dimer:GlcNAc**	–80.0 ± 0.0	–73.3 ± 0.78	–6.68 ± 0.79	5.89 ± 0.32	*N* = 1
**CVN-E41A:GlcNAc**	–78.9 ± 1.6	–71.85 ± 2.05	–7.06 ± 0.44	2.5 ± 2	4.73 ± 1.85

### Monomeric CVN-E41A Bound GlcNAc

Herein, CV-N was expressed with a single-point mutation E41T or as monomeric variant CVN-E41A, because of this site with alanine having been shown to reveal low effects on dimannose binding energies in a five-site mutated fold of the stabilized [P51G]CV-N molecule^29^ that was diminished in binding at the low-affinity binding site.^16,31^ The lectin interaction with peptide residues was enhanced or reduced by replacing a single, or two, disulfide bond(s) by ionic residue pairs (Glu and Arg) at positions E58 and R73 in CVN2, resulting in binding of dimannosylated peptide (DM) by double-mutant C58E-C73R (CVN2L0-V5) (Figure S2C) with high polar interactions.^12^ Although E41T may not be involved in electrostatic interactions with the reducing mannopyranose ring, but impaired carboxylation of glutamic acid,^31,32^ it did not fit the two-sets of site binding model. CVN-E41T showed binding to GlcNAc and bound with nanomolar affinity at the modified site (Figures S3B and S4 and Table S1). CVN-E41T recovered binding of GlcNAc with nanomolar *K*_d_ at a stoichiometry ratio of 1:1 (Figure S4 and Table S1), like the mutant dimer-bound GlcNAc (Figure 2C and Table 2), and CVN2 bound the same ligand with nanomolar *K*_d_ when possibly forming in solution nonprecipitated clusters between protein and sugar molecules (Table 2). The binding stoichiometry was calculated from a simplex fit. Thus, specific binding with nanomolar *K*_d_s was shown for the binding-site variant CVN-E41T (Figure S4), avidity of CVN2 to GlcNAc (Table 2), and binding of the monomeric variant CVN-E41A (Figure 2D) to GlcNAc (Figure 2E and Table 2).

### The Binding with GlcNAc Was Selected by the Dimer of CV-N

In comparison, CV-N binding to GlcNAc, the *N*-linked glycan unit in *N*-glycosylation sites that is also part of bacterial cell walls with beta-linked bonds to *N*-acetylmuramic acid (MurNAc), was rather unspecific and measured in the micromolar range (Figure S1B). A more selective binding of GlcNAc was therefore addressed by determining the *K*_d_ of sugar-binding sites on the dimer and investigating the effect of residue Glu41 in the high-affinity binding pocket. The sugar unbound state of domain-swapped CVN2L0 showed that Glu41 was hydrogen bonding with Gln50 and Lys48 (Figure S2B). The unmodified dimer CVN2L0 bound the DM with two different types of sites^12^ at a *K*_d_ of 3.94 μM (Figure S2D) and GlcNAc with multiple sites (Figure 2B and Figure S2E). The mutant dimer expressed from a Gibson assembly interacted with ManNAc with a *K*_d_ of 110 μM (Figure S2F), but no binding to ManNAc was analyzed for CVN-E41T (Figure S3A).

**Figure 3:**
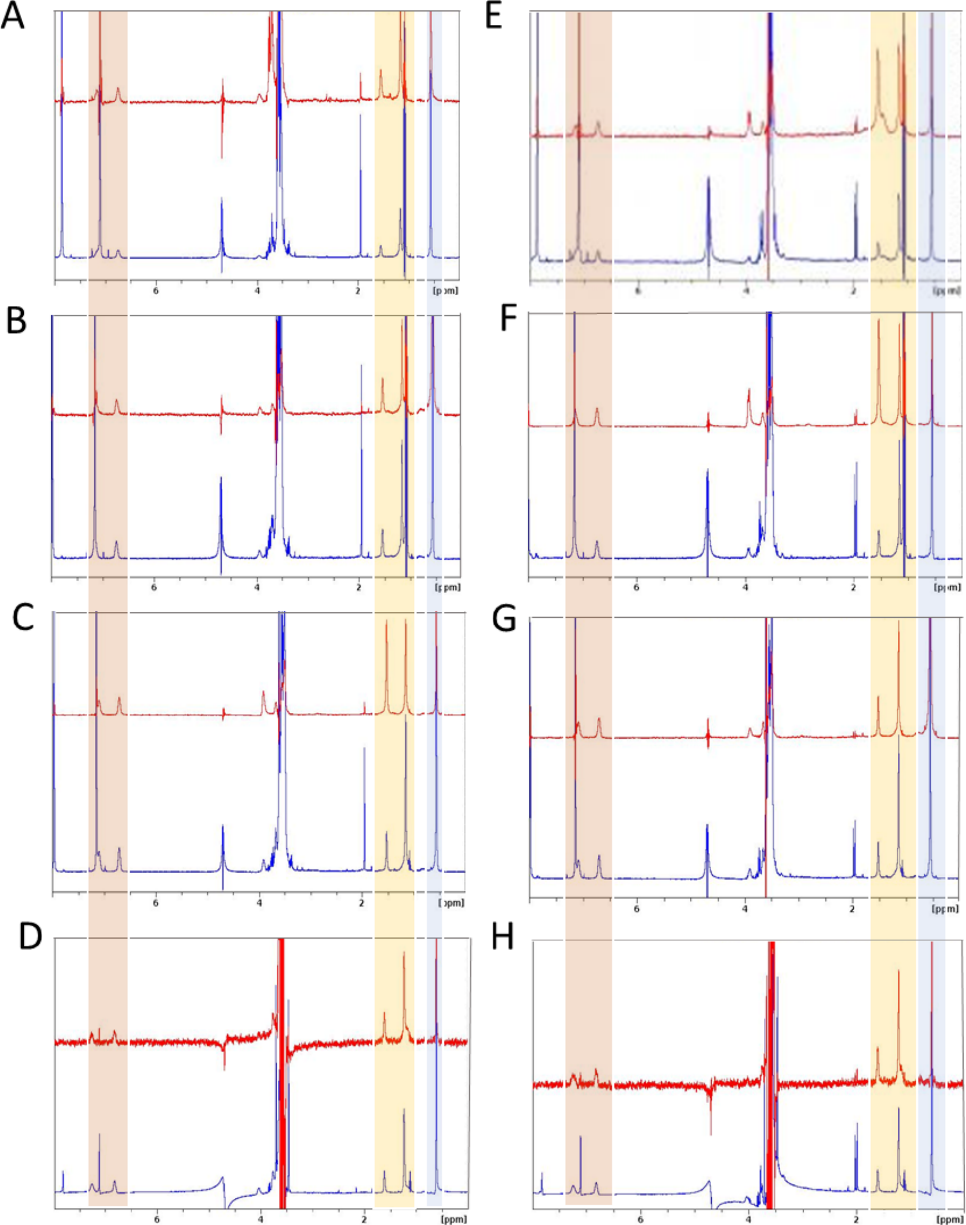
(A–D) Interaction between CVN, CVN-E41T, CVN2, mutant dimer, and GlcNAc. Bottom trace: ^1^H NMR of GlcNAc in deuterated phosphate buffer pH = 7.4 (*T* = 298 K). Upper trace: STD-NMR experiments in the presence of (A) CVN, (B) CVN-E41T, (C) CVN2, and (D) mutant dimer. (E–H) Interaction between CVN, CVN-E41T, CVN2, mutant dimer, and ManNAc. Bottom trace: ^1^H NMR of ManNAc in deuterated phosphate buffer pH = 7.4 (*T* = 298 K). Upper trace: STD-NMR experiments in the presence of (E) CVN, (F) CVN-E41T, (G) CVN2, and (H) mutant dimer. Orange, yellow, and blue bars highlighting the peaks of the amide group: orange (−NH), yellow (−COCH_3_), and blue (−CH_3_).

Using multidimensional heteronuclear NMR spectroscopy, hydrogen bonds were determined to be directed to C-2 on the terminal mannose unit of disaccharides, C-3, C-4, and C-6.^33^ The carboxylate of CV-N Glu41 located on β-strand 4,^15^ the hydroxyl group of Ser52 located in the four-amino acid linker region, and the amide and carboxylate groups of Asn53 and Glu56 presented by helical structures were forming hydrogen bonds with the dimannose in the cleft of the high-affinity site.^6,13,15^ Among these, Glu41 and Ser52, both forming a hydrogen bond with the hydroxyl group on C-2, and Gln78 have been replaced by Ala92, Gly2, and Gly27 in the low-affinity domain. Asn53 and Glu56 were found contacting with the hydroxyl group on C-4 (and Glu56 with C-6) on the terminal mannose unit and the amine-groups of Arg76 were directed to the linked mannose unit.^15^ Therefore, STD-NMR of GlcNAc^34^ showed interaction with monomeric CV-N, CVN-E41T, and dimeric CVN2 (Figure 3A–C) and hydroxyl groups of ManNAc forming hydrogen bonding with CV-N (Figure 3E) and CVN-E41T (Figure 3F). The free acetyl group of ManNAc was recognized in *cis*/*trans* conformation by the monomeric proteins CV-N and CVN-E41T (Figure 3E,F, −COCH_3_ delta = 1.5–1.8 ppm) and of GlcNAc by the respective WT dimer (Figure 3C). An alkyl group was shown in the STD-NMR spectrum of both ligands GlcNAc and ManNAc (−CH_3_ delta = 0.6–0.7 ppm). The proton (H, delta = 3.9 ppm) of the hydroxyl group of GlcNAc^35^ on C-6 is speculated to be involved in the binding with CVN2 (Figure 3C). Due to lower numbers of functional binding sites on mutant E41T shown in the binding of the carbohydrates via ITC, the isomers of the ligand ManNAc were detected comparingly (Figure 3F), but a selection and different ratio of isomers in binding ManNAc was given for the mutant dimer (Figure 3G), and in the spectra of GlcNAc in the presence of mutant monomer and dimer (Figure 3B,D). CVN2 interacted with isomeric GlcNAc. The amine group was transferred into the STD-NMR spectra (−NH, delta = 7.0 ppm) upon interaction with either GlcNAc,^36^ or ManNAc (Figure 3D,H, −COCH_3_ delta = 1.5 ppm; −CH_3_ delta = 0.6–0.7 ppm).

Furthermore, the glycosylation site on a chemically synthesized DM was introduced on a single triazole-linkage to glycine (Figure S2A). Binding of this peptide, mimicking part of the HA head domain, was measured with a *K*_d_ of 10 μM using SPR, suggesting sugar–lectin interactions at the ratio of 1:1 with only the low-affinity carbohydrate binding site.^12^ The specificity for carbohydrate-binding, however, and binding to a variety of mannose-containing moieties was supported by NMR titration and solution structure analyses.^5,7,8,14,15^ Taken together, the binding mechanism by which dimeric CVN2 revealed virus neutralization capacity of the viral spikes, was binding by two high-affinity pockets (in addition to two low-affinity carbohydrate-binding sites, PBD ID: 3S3Y, Figure S2B),^9,11^ or binding stoichiometry of two CV-N per spike protein.^10^ Contrarily, Feinberg and colleagues created a functional mimic of the asialoglycoprotein receptor from hepatocytes by incorporating conserved amino acids from the carbohydrate-recognition domain (CRD) into the homologous galactose-binding derivative of serum mannose-binding protein. The pH dependence of ligand binding was changed to become more like that of the receptor, and the modified CRD displayed selectivity for binding of *N*-acetylgalactosamine compared with galactose,^37^ demonstrating the relevance of binding-site studies.

Most studies on the binding of CV-N focused on the binding to trimeric virus envelope glycoproteins with multiple binding sites to block virus internalization and the high-mannose type of N-glycans recognized by dimeric CVN2, but no mechanism for the binding of GlcNAc has been described. NICTABA from *Nicotiana tabacum* and *Urtica dioica* agglutinin (UDA) were two antiviral lectins, which bound mannose moieties on gp120 and GlcNAc and were discovered for their GlcNAc-specific binding activity.^22^ UDA was isolated from the rhizome of the stinging nettle (*U. dioica*) as a complex mixture of isolectins with commonly two hevein-like domains binding tri-*N*-acetylchitotriose, a trimer of GlcNAc, and immunomodulatory and antiviral activity against SARS-CoV-2.^38^ Further investigation of these lectins in comparison with CVN2 and binding-site variant CVN-E41T will address the potential simultaneous binding of GlcNAc and one or more *N*-glycans on viral spikes for measuring site-specific binding in immunosorbent assays. Cross-reactivity of CV-N binding with sialic acid has not been reported yet,^10^ nor high-affinity binding measured between ManNAc and CVN2 (this study), nor chitobiose and CV-N.^7^

In conclusion, CV-N’s binding specificity to high-mannose glycans was confirmed by the interaction of CVN2 with a chemically synthesized and dimannosylated HA peptide mimetic rather than binding to structurally functional glycans. Like the mannosylated residues, ManNAc was recognized by both types of carbohydrate-binding sites on each protomer but was bound by dimeric CVN-E41T at higher micromolar concentrations than GlcNAc. The binding was compared with CVN2 binding to GlcNAc, indicating that the monosaccharide conformation on position C-2 and alkyl-groups of lectin residues directed into the binding pocket are relevant for its carbohydrate binding via the CV-N molecule. CVN-E41A also bound to GlcNAc. Amide derivatives of the tightly bound and biologically relevant GlcNAc achieved nanomolar binding affinity and 1:1 binding stoichiometry to CVN-E41T, making this sugar–lectin interaction comparably stronger than the sugar binding observed for other GlcNAc-dependent lectins.

## Supporting Information Available

(Figure S1) CV–N binding to ManNAc and GlcNAc using ITC; (Figure S2) CVN2 binding to dimannosylated peptide and GlcNAc; (Figure S3) mutant CVN-E41T binding to ManNAc and GlcNAc; (Figure S4) ITC data for binding of monomeric CVN-E41T with GlcNAc, and respective binding constants (Table S1), and binding constants of CV–N and CV–N variants to monosaccharides (Table S2 two-sets of sites model) (PDF)

## Accession Codes

CV–N: P81180 (Cyanovirin-N isolated from *Nostoc ellipsosporum*)
