# Supplementary material for: Cyanovirin-N Binding to N-Acetyl-d-glucosamine Requires Carbohydrate-Binding Sites on Two Different Protomers
Source: Biochemistry. 2024 Apr 9;63(10):1270–7. doi: 10.1021/acs.biochem.4c00113 (PMC11112747; doi:10.1021/acs.biochem.4c00113)
Supplement: Supplementary file 1 — bi4c00113_si_001.pdf [file bi4c00113_si_001.pdf]

Supporting Information

Cyanovirin-N binding to N-acetyl-D-glucosamine requires carbohydrate-binding sites on two different protomers

Irene Maier[1,2*], Georg Kontaxis[3], Christian Zimmermann[4], and Christoph Steininger[2]

[1]Department of Environmental Health Sciences, Fielding School of Public Health, University of California, Los Angeles, 650 Charles E. Young Dr. South, Los Angeles, CA-90095, Unites States

[2]Department of Internal Medicine I, Medical University of Vienna, Waehringer Guertel 18-20, A-1090, Vienna, Austria

[3]Department of Computational and Structural Biology, Max Perutz Laboratories, University of Vienna, Campus Vienna Biocenter 5, 1030, Vienna, Austria

[4]Institute of Chemical, Environmental and Bioscience Engineering, TU Wien, Gumpendorfer Strasse 1a, 1060 Wien, Austria.

Content:

Figure S1: CV-N binding to ManNAc and GlcNAc using ITC

Figure S2: CVN2 binding to dimannosylated peptide and GlcNAc

Figure S3: Mutant E41T binding to ManNAc and GlcNAc

Figure S4: ITC data for binding of monomeric CVN-E41T with GlcNAc

Table S1: Thermodynamic binding parameters for mutant CVN-E41T

Table S2: Binding constants of CV-N and CV-N variants to monosaccharides (two-sets of sites model).

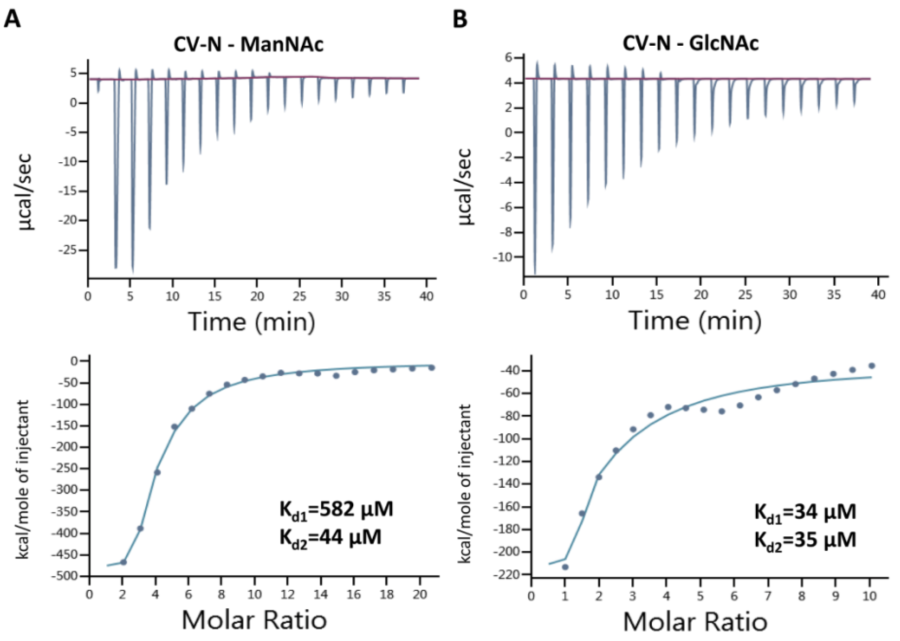

Figure S1. Cyanovirin-N binds to both ManNAc and GlcNAc with a stoichiometry of 3 CV-N: 1 ManNAc and 1 CV-N: 1 GlcNAc, respectively. Isothermal titration calorimetry (ITC) data showing the binding of CV-N to these monosaccharides. **(A)** Thermogram and calculate binding to ManNAc. **(B)** Thermogram and calculate binding to GlcNAc. Calculated energies of interaction, affinities, and stoichiometry for CV-N binding and other variants to either of the monosaccharides, or ManNAc, are summarized in Table S1.

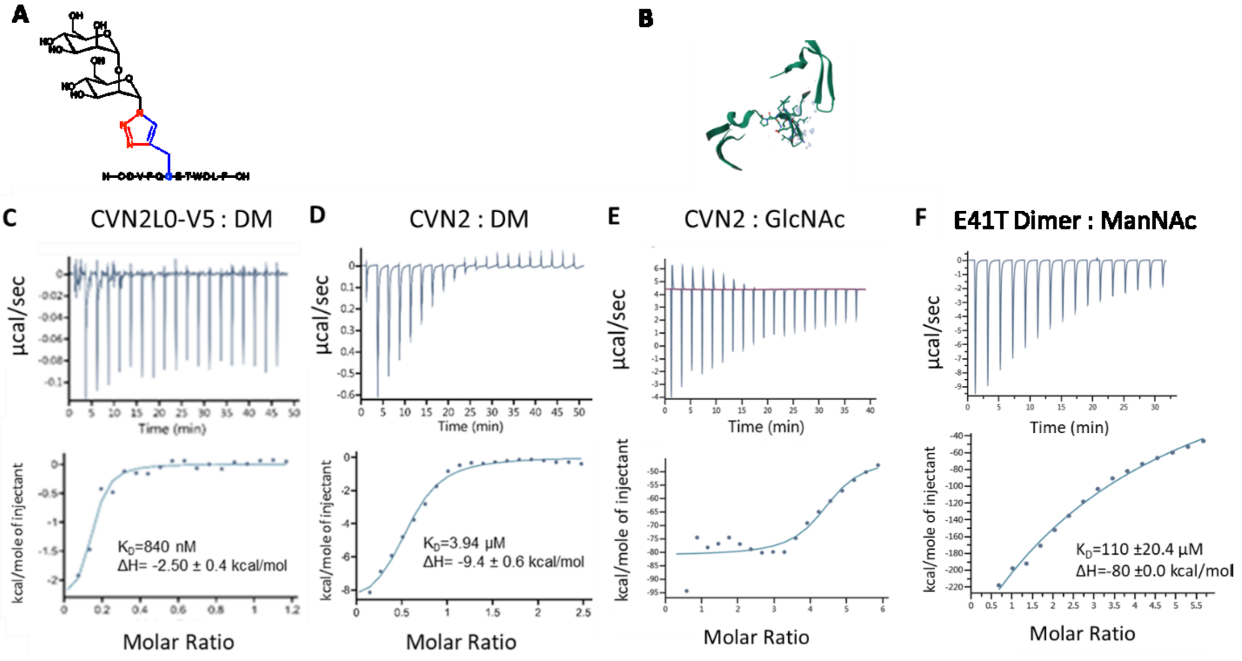

Figure S2: Binding of (A) dimannosylated peptide to CVN2 and mutant CVN2 and (B) CVN2 3D X-ray structure (PDB ID: 3S3Y). (C) ITC data showing the binding of mutant CVN2L0-V5 to dimannosylated peptide (DM).[1] **(D)** ITC showing CVN2 binding to DM. Thermograms are shown, and binding curves are fitted to the one-set of sites binding model. T=298K. Heats of binding were measured as delta (Δ)H. **(E)** CVN2L0 binding to GlcNAc. Thermogram is shown and binding curve fitted to the two-sets of sites binding model. Data for E are summarized in Table S2. **(F)** Mutant E41T dimer binding to ManNAc is calculated based on N=2, ligand in the cell analysis.

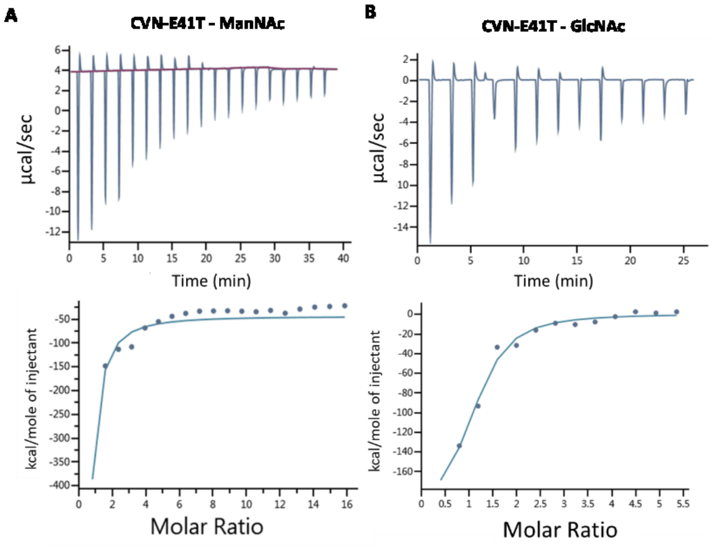

Figure S3: ITC data for monomeric CVN-E41T with **(A)** ManNAc and **(B)** GlcNAc. Thermograms are shown, and binding curves are fitted to the one-set of sites binding model. T=298K **(A-B)** Binding constants are summarized in Table S1.

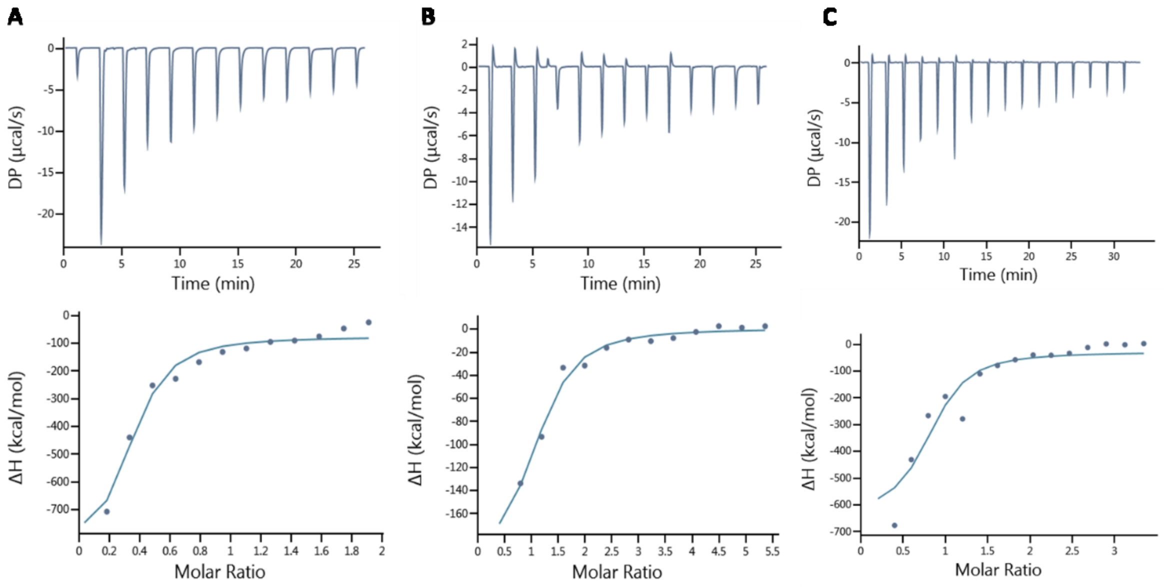

Figure S4: ITC data for binding of monomeric CVN-E41T with GlcNAc. **(A-C)** Thermograms are shown, and binding curves are fitted to the one-set of sites binding model for three biological replicates at a ligand concentration of 6µM. T=298K.

Table S1. Thermodynamic binding parameters for mutant CVN-E41T.

	Enthalpy *ΔH (kcal/mol)*	*Entropy* *TΔS (kcal/mol)*	*Free Energy* *ΔG(kcal/mol)*	*Affinity* *Kd [nM]*	*Stoichiometry*
E41T:ManNAc	-80.0 ±0.0	-74.2 ±0.7	-5.9 ±0.86	n.a.	2.04 ±0.468
CVN-E41T:GlcNAc	**-65.5**	**-57.1**	**-8.37**	**728**	**1**
**CVN-E41T:GlcNAc**	**-80.0**	**-71.3**	**-8.73**	**397**	**1**
**CVN-E41T:GlcNAc**	**-80.0**	**-71.3**	**-8.70**	**418**	**1**

Table S2. Thermodynamic binding parameters of monomer CV-N, dimeric CVN2 and CVN-E41T to ManNAc and CV-N and CVN2 to GlcNAc calculated from the two-sets of sites model.

	ManNAc			GlcNAc	
	CV-N	CVN2	CVN-E41T	CV-N	CVN2
KD1 [M]	582e-6	5.2e-6	712e-6	34e-6	108e-6
KD2 [M]	44e-6	2.6e-9	17.6e-6	35e-6	2.84e-6
N1	1	1	4	1	1
N2	3	4	4	1	4
ΔH1 (kcal/mol)	-59.0	-77.0	-34.8	-80.0	1.79
ΔH2 (kcal/mol)	-80.0	-79.2	-80.0	-80.0	-40.1
-TΔS1 (kcal/mol)	54.6	69.8	30.5	73.9	-7.21
-TΔS2 (kcal/mol)	74.1	67.5	73.5	73.9	32.5

1. Schilling, P.E.K., Dragosits, M., Schiestl, R. H., Becker, C.F.W., Maier, I., Mannosylated hemagglutinin peptides bind cyanovirin-N independent of disulfide-bonds in complementary binding sites. RSC Adv, 2020. 10(19): p. 11079-87. doi: 10.1039/d0ra01128b
